# C-terminal motif prediction in eukaryotic proteomes using comparative genomics and statistical over-representation across protein families

**DOI:** 10.1186/1471-2164-8-191

**Published:** 2007-06-26

**Authors:** Ryan S Austin, Nicholas J Provart, Sean R Cutler

**Affiliations:** 1 Department of Cell & Systems Biology, University of Toronto, 25 Willcocks St., Toronto, ON. M5S 3B2, Canada; 2 Center for Plant Cell Biology (CEPCEB), Department of Botany and Plant Sciences, University of California, Riverside, CA 92521, USA

## Abstract

**Background:**

The carboxy termini of proteins are a frequent site of activity for a variety of biologically important functions, ranging from post-translational modification to protein targeting. Several short peptide motifs involved in protein sorting roles and dependent upon their proximity to the C-terminus for proper function have already been characterized. As a limited number of such motifs have been identified, the potential exists for genome-wide statistical analysis and comparative genomics to reveal novel peptide signatures functioning in a C-terminal dependent manner. We have applied a novel methodology to the prediction of C-terminal-anchored peptide motifs involving a simple z-statistic and several techniques for improving the signal-to-noise ratio.

**Results:**

We examined the statistical over-representation of position-specific C-terminal tripeptides in 7 eukaryotic proteomes. Sequence randomization models and simple-sequence masking were applied to the successful reduction of background noise. Similarly, as C-terminal homology among members of large protein families may artificially inflate tripeptide counts in an irrelevant and obfuscating manner, gene-family clustering was performed prior to the analysis in order to assess tripeptide over-representation across protein families as opposed to across all proteins. Finally, comparative genomics was used to identify tripeptides significantly occurring in multiple species. This approach has been able to predict, to our knowledge, all C-terminally anchored targeting motifs present in the literature. These include the PTS1 peroxisomal targeting signal (SKL*), the ER-retention signal (K/HDEL*), the ER-retrieval signal for membrane bound proteins (KKxx*), the prenylation signal (CC*) and the CaaX box prenylation motif. In addition to a high statistical over-representation of these known motifs, a collection of significant tripeptides with a high propensity for biological function exists between species, among kingdoms and across eukaryotes. Motifs of note include a serine-acidic peptide (DSD*) as well as several lysine enriched motifs found in nearly all eukaryotic genomes examined.

**Conclusion:**

We have successfully generated a high confidence representation of eukaryotic motifs anchored at the C-terminus. A high incidence of true-positives in our results suggests that several previously unidentified tripeptide patterns are strong candidates for representing novel peptide motifs of a widely employed nature in the C-terminal biology of eukaryotes. Our application of comparative genomics, statistical over-representation and the adjustment for protein family homology has generated several hypotheses concerning the C-terminal topology as it pertains to sorting and potential protein interaction signals. This approach to background reduction could be expanded for application to protein motif prediction in the protein interior. A parallel N-terminal analysis is presented as supplementary data.

## Background

The carboxy tails of proteins are frequent sites of post-translational modification, protein-protein interaction domains and sub-cellular protein sorting motifs. This is presumably due to a high-kinetic cost in burying the termini within the interior of the protein; leaving the head and tail regions of many proteins exposed to the cytoplasm and free to engage in static or dynamic biochemical interactions [1]. Although a variety of protein domains have been characterized to preferentially or even exclusively occur within the terminal regions, a class of signatures has been found to be effectively dependent upon their proximity to the C-terminal end for proper function. Members of this class of motifs include: the peroxisomal PTS1 signal (SKL-COOH), the ER retention signal (K/HDEL-COOH), the ER retrieval signal for membrane bound proteins (KKxx-COOH) and the protein C-terminal prenylation motif (Caxx-COOH). These motifs appear as a frequent sorting strategy in diverse protein groups and are mostly conserved throughout eukaryotes [1-5]. Since such signals are often critical to proper function, they are likely to be highly resistant to selective pressure and therefore evolutionarily conserved in numerous protein classes and species genomes. This conservation should be detectable in whole genome analysis as a statistical over-representation of motif derived tripeptides against a background of tripeptide expectation by chance alone.

In general, protein motif prediction can be divided into two basic approaches, the *a priori *mapping of experimentally verified motifs to novel unannotated sequences (scanning) and the *ab initio *identification of potentially novel motifs without any prior knowledge of motif structure. Over the past decade, rapid advances in high-throughput proteomics and a large body of literature detailing the structure and function of numerous proteins in many species, have focused protein motif prediction on the annotation of novel sequences using motif scanning from an *a priori *collection of protein domain knowledge in the literature [6,7]. Effective sequence alignment algorithms and an abundance of coding sequence data have allowed for the effective identification of conserved sequence domains among orthologous proteins, limiting the need for *ab initio *protein motif prediction methods. Nevertheless, *ab initio *prediction methods are likely to play a significant role in our completion of a comprehensive protein domain grammar. In addition to *ab initio *prediction, integrative methods have applied protein-protein interaction maps, crystallography data, NMR results and amino acid frequencies to the prediction of novel functional domains in diverse classes of proteins [8-11]. *Ab initio *prediction of novel protein motifs from primary sequence using heuristical approaches, enumerative measures, orthologous sequences, functional annotation and statistical over-representation have all been explored using an integrative framework [12-15].

Methods that assay sequence statistical over-representation apply chi-squared, p-values or z-scores to nmer frequencies, most often in association with one or more expectation values or a randomized background model. The reasoning behind such approaches is that motifs of critical functional significance are expected to be more highly conserved than benign stretches of primary sequence free from selective pressure. Thus short sequence stretches of critical function should exhibit higher statistical frequencies than non-critical regions more tolerant to changes and variation in residue make-up. Unfortunately, as a low signal-to-noise ratio is a frequent problem in sequence analysis, such studies require careful selection of a background model that will optimally reduce this biological 'noise' [11,15]. Bayesian inference, sequence randomization and the use of hidden Markov models have all been explored to this effect. However, those approaches that most closely model the biological background appear to be the most effective in reducing the false positive rate [16]. In addition to the complications of motif degeneracy, variability in the positioning of individual motifs along the length of genetic sequences introduces computationally expensive considerations into the analysis. Hence, the ability to define a biologically relevant reference point from which to examine sequence prevalence can greatly simplify statistical calculations [17]. This has been applied to the prediction of transcription factor binding sites in relation to the transcription start site as well as in the examination of both nucleotide and peptide frequencies in relation to the protein termini [14,18-21].

Statistical studies of nucleotide and peptide frequencies in the C-terminus of eukaryotic genomes have revealed non-random nucleotide, amino acid and short peptide biases [17-20,22,23]. In 2003, Chung et al. tallied the frequencies of C-terminal 3mers and 4mers in several eukaryotic genomes to show that known targeting signals ranked highly in several species [22]. In that same year, Gatto & Berg likewise compared C-terminal tripeptide frequencies to a shuffled proteome to identify known motifs as over-represented in several eukaryotic proteomes [18]. However, efforts to increase the low signal-to-noise ratio inherent in such analyses have not been fully explored and a high-confidence snapshot of biologically relevant C-terminal topology has yet to be determined. We therefore reasoned that the exploration of randomized sequence background models along with additional data that incorporates protein family information and comparative genomics could reduce background levels enough to accurately depict a collection of eukaryotic conserved C-terminal anchored protein motifs (CTAMs). As C-terminal sequence homology between common members of large protein families has been postulated to heavily contribute to individual nmer counts in frequency calculations [18], our test statistic (z-score) tallied any multiple tripeptide counts arising from members of a common gene family as a single instance. This effort was able to identify a collection of eukaryotic-conserved statistically over-represented C-terminal tripeptides (SOCTs), many of which correspond to known C-terminally anchored sequences, as well as several other novel and intriguing motif patterns within the C-terminal biology of the 7 species examined.

## Results

**Figure 1:**
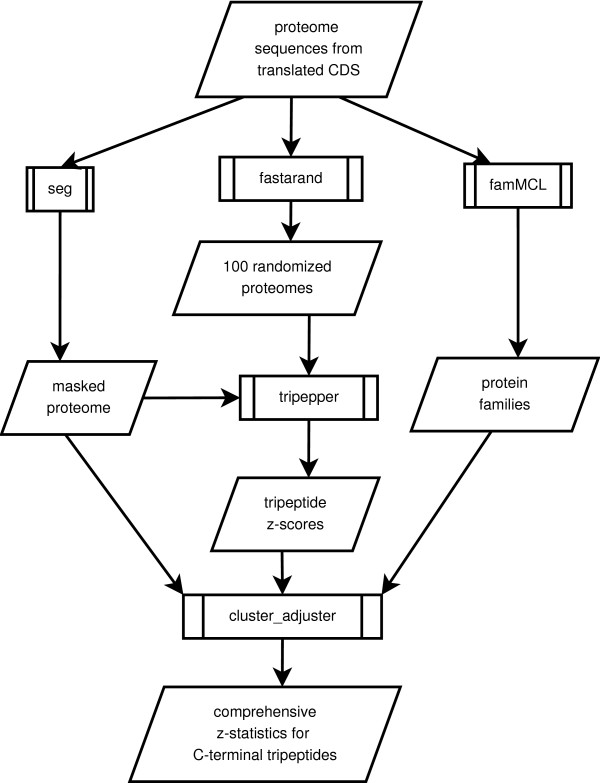
**Flowchart of the SOCT pipeline**. A combination of filters and pre-processing was performed against individual proteomes to obtain a comprehensive set of z-statistics for each possible tripeptide at all positions from the C-terminal end to 100 residues in from the C-terminus. Programs and scripts for data analysis are represented as barred boxes, while resulting datasets are depicted as polygons.

We applied a novel methodology to the prediction of biologically active sequence motifs at the C-terminus of 7 eukaryotic genomes (*A. thaliana, O. sativa, S. cerevisiae, C. elegans, D. melanogaster, M. musculus *and *H. sapiens*). Generally, our methodology applied a penalized z-statistic that disregarded tripeptide frequencies arising from simple sequences or from C-terminal homology among members of protein families. Comparative genomics of SOCT frequency between species pairs and across all species was then used to filter for C-terminal protein motifs potentially involved in generalized protein biology roles such as protein sorting and post-translational modification (see Fig 1).

The general implementation of our method for each proteome is as follows:

1) generate a randomized background of c-terminal peptide frequencies from proteome sequence

2) mask low-complexity sequences within the c-terminal regions

3) generate comprehensive position-specific z-statistics for all possible tripeptides occurring at positions from -3 to -100 residues in from the carboxy terminus.

4) determine gene family clusters for the proteome

5) adjust z-scores to exclude duplicate tripeptide counts arising from within individual gene families.

**Figure 2:**
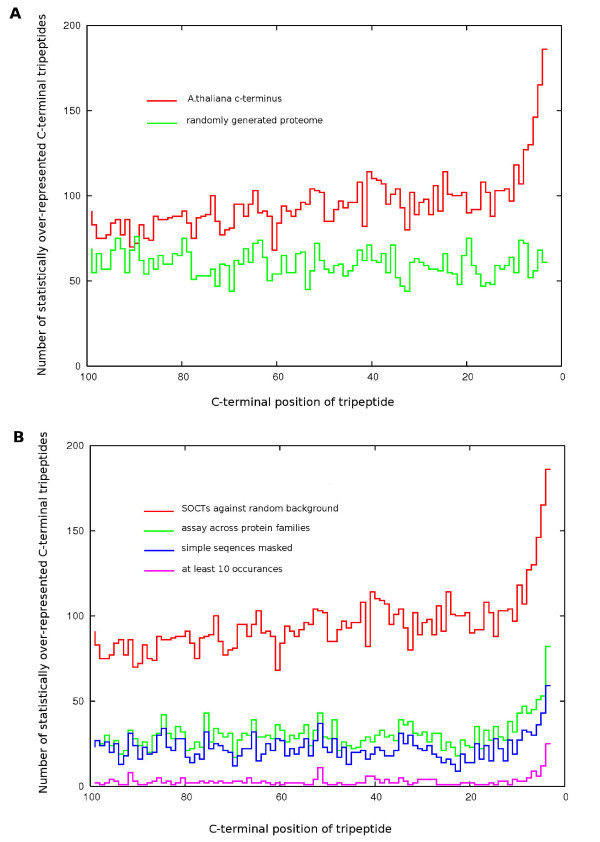
**Position-specific abundance of SOCTs in *A. thaliana***. Graphical depictions of the number of statistically over-represented C-terminal tripeptides (z ≥ 3) occurring in the C-terminal region (-3 to -100). **A**. The unfiltered assessment of statistical over-representation in the C-terminus, as compared to a randomized data set control. **B**. The reduction in site-specific SOCT abundance after successive rounds of filtering measures including sequence masking, protein family adjustment and the stipulation of at least 10 occurrences for each SOCT.

Initial analysis, performed without sequence masking or protein family filtering, reconfirmed the strong terminal bias in tripeptide composition seen by Gatto & Berg [18]. This bias has also been observed at the levels of amino acid [19,24], nucleotide and codon composition [20] and decamer peptides [17]. Our results extend the confirmed presence of a terminal tripeptide bias to include the genomes of *O. sativa*, *C. elegans*, *D. melanogaster *and *M. musculus *[see Additional file 7 for the N-terminal data set]. It would appear that this composition bias exists at all levels of analysis in all species from bacteria to higher eukaryotes. In this study, we represent the terminal bias by the presence of a disproportionate amount of 'statistically over-represented C-terminal tripeptides' (SOCTs) in the extreme carboxy terminal positions (z ≥ 3.0, see Fig 2).

### Genomic data and sequence pre-filtering

Predicted protein databases for each species were downloaded in fasta format from NCBI with the exceptions of *A. thaliana*, which was obtained from TAIR, and *O. sativa*, which was downloaded from TIGR. As the *O. sativa *genome contains more than 17% transposable elements, these sequences represented a high potential for skewing tripeptide counts unfavourably and are recommended for removal from such whole genome analyses [18]. The *O. sativa *dataset was therefore, pre-filtered to remove all sequences annotated as a transposable element prior to the analysis. This measure dramatically reduced the level of background noise in our results. This is because the abundance of retro-element-type sequences in rice can not only obfuscate the biologically relevant background tripeptide frequencies, but result in numerous clusters of transposon derived gene families in our clustering efforts. These 'junk clusters' artificially inflate tripeptide counts and their respective z-scores. As rice was the only dataset to possess such an exceptionally large percentage of annotated retroelements, it was the only proteome pre-filtered in this manner [see Additional file 2].

Another confounding factor is simple sequences, which are stretches of low complexity residue repeats of a presumably benign or possibly structural function, and which are known to skew sequence statistics [25]. Masking of these sequences prior to statistical analysis is a frequent strategy in sequence searching algorithms (e.g. *BLAST*) [26]. Due to the presence of numerous simple sequence-like tripeptides with significant scores in our preliminary work and in prior studies [18,22], *seg *filtering was applied to each species proteome prior to obtaining individual tripeptide counts and comparison against the randomization model. It should be noted that the randomly generated fasta sets were not pre-filtered with *seg*. This measure results in greater background averages for simple-sequence-like tripeptides and translates into an increase in the stringency against such tripeptides via lower z-scores. Overall, these measures removed several simple-sequence-like tripeptides from our significant results and succeeded in lowering observed SOCT abundance levels slightly (see Fig 2).

### Background randomization models

Our approach adopted the strategy of genome randomization for assessing expectant tripeptide frequencies. Each respective species proteome was randomized 100 times in order to obtain a frequency distribution for each possible tripeptide at all positions from the C-terminal positions of -3 to -100. The expected mean and standard deviation values derived from these random sets were compared to observed tripeptide counts in the actual proteome in order to derive a position-specific tripeptide z-score. Three peptide randomization models were tested for their ability to affect the level of position specific SOCT abundance. Briefly, peptide sequences of equal length to every protein in the proteome were iteratively generated using a program *fastarand *written in the C programming language. The randomization models included: 1) randomization based on amino acid frequencies for the entire proteome, 2) shuffling amino composition in individual proteins, and 3) the sampling of tripeptide content in each protein, with the potential for the resampling of any particular tripeptide in the sequence. Methods 2 and 3 proceed iteratively on a protein-by-protein basis using the composition of each protein in the proteome to generate randomized versions of each sequence. Of the three methods, the third was chosen as the model for further filtering, as it resulted in the largest reduction in overall frequencies of statistically significant tripeptides at each position in the terminal tail [see Additional file 1]. Model 2 was the next best method at reducing background noise, with model 1 being least effective.

### Protein family prediction

In their 2003 analysis of C-terminal tripeptide frequencies, Gatto & Berg identified several over-represented tripeptides as arising from homology within the C-termini of large protein families [18]. In such instances, the tallies of individual tripeptides could be exaggerated beyond what is expected by chance. Since our objective was to predict general protein targeting or PTM signals occurring among many diverse proteins, the exclusion of tripeptide counts arising from large homologous protein families was used to lower the high position-specific SOCT frequencies seen in our unfiltered results (see Fig 2), an approach not taken by Bahir & Linial [23]. This evaluation of tripeptide frequencies at the level of the protein family instead of the individual protein then allows for the specific assaying for such signatures that occur as genome-wide over-represented signals due to generalized structural or functional requirements in C-terminal biology.

To determine tripeptide significance levels across protein families, each proteome was first clustered into gene families using our short UNIX shell script *famMCL*. *famMCL *performs: 1) an all-against-all *BLASTP *comparison between proteins in a proteome; 2) parses the *BLAST *output for bitscore values (cutoff: E < 1e-10); 3) submits an MCL matrix of bitscores to the *Markov Clustering Algorithm *(*MCL*) [27]; and 4) renders the *MCL *output into a user readable list of gene families. The data output format and interface to the *MCL *algorithm was modeled after Enright et al.'s work of 2002, using bitscores in place of E-values and adding in an automatic all-by-all blasting routine [28]. Comprehensive bitscore parsing of *BLASTP *output provides for a straightforward implementation with more complete and accurate similarity matrices and overall better cluster approximations. This strategy is used in both the *MCL *implementation of gene family prediction *mclblast *as well as in the prediction of clusters of orthologous genes in *orthoMCL *[29].

Clustering gene families in this manner, we obtained an average of almost 4000 gene families with 2 or more members for each of the 6 higher eukaryotes, with *C. elegans *possessing the fewest clusters at 2725, and *O. sativa *possessing the most at 5452. *S. cerevisiae*, in accordance with its smaller genome size, possessed considerably fewer predicted protein families at only 749 clusters with 2 or more members [see Additional file 5]. In each case, the number of 2 member clusters accounted for approximately 50% of the total cluster number, with *S. cerevisiae *having the most 2 member clusters (68%) and *A. thaliana *the least (42%). When gene familes of at least 10 members were considered, the number of gene clusters dropped to 80 and 109 for the plants, 31 and 36 for the lower animals, 52 and 68 for the mammals and 3 for yeast. Individual protein identifiers from within separate clusters were then appended with their annotations to confirm consistencies in functional annotation and to ensure that the algorithm was working correctly (data not shown).

The resulting clusters for each species were then used to assess tripeptide over-representation across protein families. Basically, all tripeptides frequencies were assessed in a manner that allowed for only a single tripeptide count to arise from within any single gene family. This measure prevents multiple tripeptide counts due to C-terminal homology in gene families from artificially inflating our tripeptide frequencies and unrealistically skewing our over-representation statistics. Overall, these efforts improved the signal-to-noise ratio considerably; as evident in a significantly reduced number of SOCTs at each C-terminal position (see Fig 2). Additionally, numerous CTAMs were now readily identifiable in the results in all species and the C-terminal biases observed could represent targeting motifs, post-translational modification signals, protein-protein interaction domains or structural tendencies in C-terminal biology such as capping and orientation strategies. This technique of assaying peptide frequencies as they pertain to protein family tendencies would appear an effective measure for the prediction of trends in biological sequence preferences at a genome-wide level and could be adapted to the prediction of protein domains in the protein interior.

### Terminal biases persist after aggressive filtering

Our analysis defined the terminal bias as a dramatic rise in the number of statistically over-represented (z ≥ 3) C-terminal tripeptides (SOCTs) in the last 15 to 20 tripeptide positions of each "C-terminome". In *A. thaliana*, the filtering of tripeptide tallies using a maximum count of 1 occurrence from separate protein families reduced background levels by approximately 70 SOCTs per position, while simple sequence masking reduced background noise by approximately 10 SOCTs per position (see Fig 2). Comparable results were seen in the other species examined. Interestingly, the ratio of extreme C-terminal SOCT count (-3) to average SOCT counts at positions proximal to the terminal region (-100 to -10) increases with each successive filtering. We believe this reflects, with each round of filtering, a progression from a terminal bias arising due to genome-wide selective pressures in C-terminal residue composition to the most functionally distinct and biologically relevant C-terminal tripeptides.

### Eukaryotic protein tails share conserved tripeptides at positions -3 and -4

Overall, our analysis identified numerous statistically over-represented C-terminal tripeptides in all species, the majority of which existed in the C-terminal bias region from -3 to -5 (see Fig 2). Specifically, the number of SOCTs occurring in each species at the extreme terminal end was: *A. thaliana*, 42; *O. sativa*, 77; *S. cerevisiae*, 108; *D. melanogaster*, 25; *C. elegans*, 90; *M. musculus*, 41; and *H. sapiens*, 45. The elevated levels of SOCTs in worm and yeast may be a result of the smaller genome sizes. It was our assumption that many of these SOCTs would be false positives and since we wished to identify sequences conserved as general biological strategies, individual intersections of SOCT totals were taken between pairs of more closely related species.

**Figure 3:**
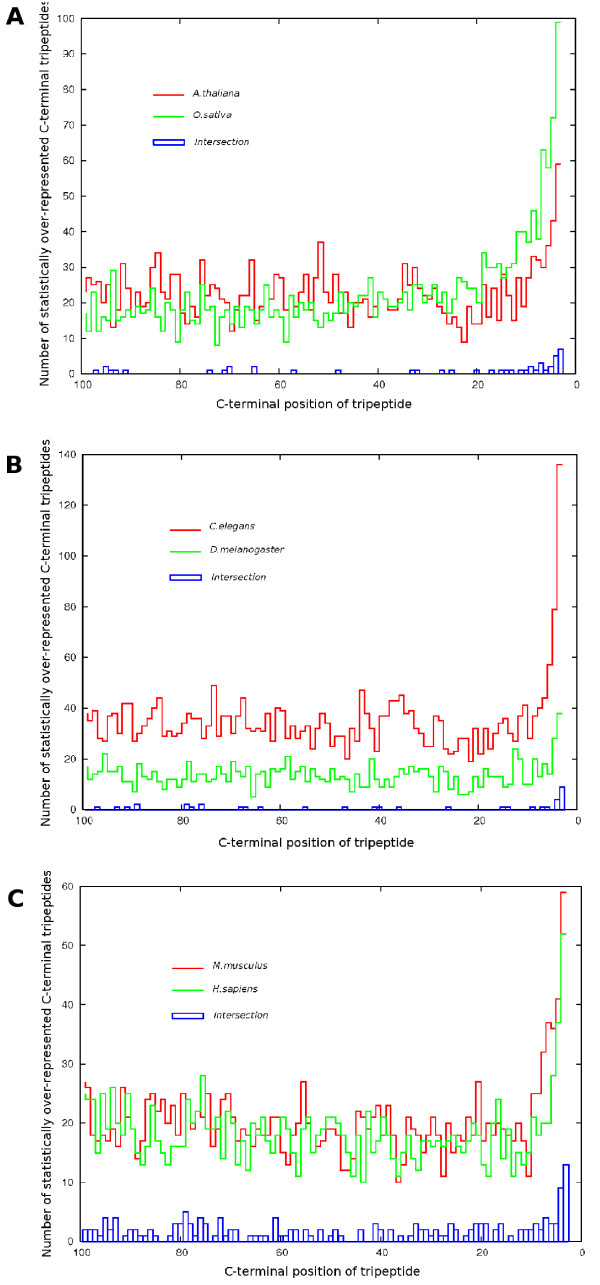
**SOCT intersections between species**. Intersections of statistically over-represented tripeptides at the C-terminus of **A. **the two plant species (*A. thaliana, O. sativa*), **B**. the two lower animals (*C. elegans, D. melanogaster*) and **C**. the two mammalian proteomes (*H. sapiens, M. musculus*). The SOCT abundance at each C-terminal position is graphed for each species with the the number of commonly occurring SOCTs between the two species depicted with blue boxes.

Using our final filtered z-scores for all species, comparisons were made in SOCT conservation between the plants, the lower animals and the mammals (see Fig 3). In each case, similar to the C-terminal bias, the number of SOCTs occurring in each species pair overlapped most frequently in the last 2 tripeptide positions (i.e. positions -3 and -4). Intersections of SOCTs between the two plant species (rice and Arabidopsis) and the two lower animals (fly and worm) showed the presence of several Caax box motifs and the canonical PTS1 consensus of SKL. The ER retention signal [HK]DEL was conspicuously absent from the plant intersections, although this was due to its under-representation in *O. sativa*. As the *O. sativa *genome is the least well annotated of all the predicted proteomes, this lack of significance for the ER retention signal is likely an artifact. Indeed, a total of 40 proteins in the *O. satvia *genome match the ER retention consensus of [KH]DEL. The DEL SOCT was also absent in the lower animals due to its lack of significance in *C. elegans*. This may represent the presence of an alternate preferred ER retention consensus motif in worms. The 2 mammalian species (human and mouse) possessed Caax motifs, several PTS1 consensus variants and the HDEL form of the ER retention signal, see Table 1, 2.

**Table 1:** SOCT intersections between plants, lower animals and mammals – z statistics. Final filtered z-statistics for 7 eukaryotic C-terminal tripeptides (z ≥ 3.0) occurring at positions -3 and -4 and intersected between the two plant species (*A. thaliana *– AT, *O. sativa *– OS), the two lower animals (*C. elegans *– CE,*D. melanogaster *– DM), and the mammals (*M. musculus *– MM,*H. sapiens *– HS). B. Species-specific lists of all SOCTs (z ≥ 3.0) at the -3 and -4 positions and occurring in at least 10 genes in each respective species proteome. This latter stipulation is provided for the sake of brevity and the reader is referred to the Additional files section for the complete data set [see Additional file 6]."

***C. elegans & D. melanogaster***			
**peptide**	**offset**	**CE z-score**	**DM z-score**
**SKL**	3	8.4	3.2
**HKY**	3	3.7	3.4
**GKK**	3	5.8	3.6
**RRK**	3	3.3	3.3
**FNF**	3	5.1	4.0
**KKK**	3	10.0	8.0
**DSD**	3	6.2	3.4
**RPW**	3	3.6	3.5
**DED**	3	3.3	3.2
**HDE**	4	4.3	5.0
**CTI**	4	4.3	6.0
**CSI**	4	6.0	3.9
**CVI**	4	6.9	3.0
			
***O.sativa & A.thaliana***			
**peptide**	**offset**	**OS z-score**	**AT z-score**

**SKL**	3	7.8	6.5
**SIM**	3	6.2	4.4
**DFM**	3	3.1	4.0
**RCC**	3	4.2	3.0
**KCP**	3	5.7	3.7
**YRY**	3	3.9	4.9
**FYS**	3	3.0	3.6
**PKC**	4	3.8	3.2
**CTI**	4	4.7	8.2
**WWW**	4	4.1	4.7
**CCI**	4	6.7	3.8
**CSI**	4	9.1	8.1
			
***M.musculus & H.sapiens***			
**peptide**	**offset**	**MM z-score**	**HS z-score**

**THL**	3	4.2	5.2
**DEL**	3	8.1	7.8
**TEL**	3	4.6	4.6
**DEF**	3	3.7	4.3
**TRL**	3	6.2	3.3
**SRK**	3	3.4	3.3
**KKK**	3	4.3	3.8
**DSD**	3	4.5	4.6
**SCC**	3	3.7	4.0
**YMW**	3	3.1	5.4
**TTV**	3	5.8	7.2
**RKK**	3	5.3	3.6
**TKL**	3	3.5	5.8
**HDE**	4	6.5	6.6
**FWW**	4	3.1	3.0
**CTK**	4	3.1	4.6
**WRP**	4	5.4	5.0
**CTI**	4	3.6	7.7
**RWT**	4	3.3	4.4
**QYN**	4	3.2	3.2
**ESE**	4	3.2	3.1
**CVI**	4	5.1	4.5

**Table 2:** Species-specific lists of all SOCTs. – Species-specific lists of all SOCTs (z ≥ 3.0) at the -3 and -4 positions and occurring in at least 10 genes in each respective species proteome. This latter stipulation is provided for the sake of brevity and the reader is referred to the Additional files section for the complete data set [see Additional file 6][Supplementary-material S6]

***AT***	***OS***	***OS***	***SC***	***CE***	***CE***	***DM***	***MM***	***HS***
ADS.3	DIF.3	CSV.4	SKL.3	FGK.3	RFF.3	SKL.3	QSR.3	THL.3
KRT.3	QLI.3	SYY.4	LKK.3	SIF.3	FEF.3	TEL.3	TAL.3	DEL.3
SKL.3	IFL.3	QFV.4	SKK.3	TRF.3	VKN.3	KSK.3	THL.3	TEL.3
DEL.3	SKL.3	KAN.4	KKK.3	PPQ.3	RKK.3	AKL.3	DEL.3	DEF.3
SRL.3	TSN.3	IEE.4	DEL.3	RSL.3	RRF.3	TKS.3	TEL.3	TKK.3
SIM.3	FED.3	HSK.4	AKK.3	QKI.3	RRR.3	GKK.3	GSC.3	TSL.3
FTS.3	KIN.3	DQE.4	LSK.3	SKL.3	KSE.3	RRK.3	SKI.3	KSN.3
RKR.3	TKN.3	IDK.4	LLK.3	TVE.3	VSS.3	LKK.3	KDI.3	TSV.3
QTL.3	CIL.3	PKK.4	KKK.4	KIN.3	DED.3	KKK.3	GQS.3	TRL.3
KQD.3	KGN.3	KII.4	HDE.4	SKK.3	IGK.3	DSD.3	SHL.3	SRK.3
KRR.3	VTS.3	YFL.4		VIN.3	RKL.3	DED.3	ESH.3	KKK.3
HSS.3	SIM.3	DYS.4		SKA.3	TNN.3	KAK.3	AAS.3	RRC.3
IFF.3	MGI.3	CSI.4		DEL.3	FSF.3	KIK.3	KTT.3	DSD.3
NQS.3	RKN.3	TTV.4		FKF.3	KRK.3	KNK.3	TRL.3	CCA.3
PSY.3	SLY.3	TNK.4		IKN.3	LFN.3	KRR.4	SNV.3	SCL.3
TRT.3	FFS.3	SSK.4		IKF.3	FGR.4	KSN.4	SRK.3	SCC.3
RRH.3	ISF.3	QIR.4		DEE.3	GTR.4		KKK.3	KKN.3
TKD.3	SYY.3	FQR.4		IEN.3	GSR.4		DSD.3	TTV.3
DSD.3	LKH.3	CVI.4		QKF.3	NLK.4		SCC.3	NHL.3
NTN.3	KNN.3	YFF.4		DDE.3	KSK.4		TTV.3	TDV.3
TSH.3	TVR.3	KRK.4		PSA.3	ISK.4		EEL.3	RKK.3
RRR.3	EIN.3	LNY.4		FFN.3	DDE.4		RKK.3	KTD.3
MSL.3	QQK.3	TSR.4		SKN.3	YLG.4		QES.3	TKL.3
FYS.3	PKY.3			KML.3	VFD.4		TKL.3	KRK.3
KPF.3	YKL.3			KKI.3	TKK.4		SFY.3	TSI.3
HDE.4	HFL.3			LGP.3	NSK.4		HDE.4	TVV.3
SNT.4	IPK.3			TQF.3	EDS.4		RKT.4	HDE.4
SRR.4	HRF.3			SRR.3	PIN.4		SSM.4	KKA.4
KKQ.4	IQV.3			KNN.3	GKK.4		FSK.4	ETV.4
CTI.4	IRS.3			QIF.3	SRK.4		KPK.4	CTI.4
RSR.4	NQN.3			IDF.3	KKK.4		KTD.4	RKI.4
DSD.4	LIN.3			SKY.3	SDS.4		ESE.4	ETS.4
CSI.4	KCP.3			PGY.3	KKS.4		ISQ.4	SCC.4
PSK.4	KKN.3			KKQ.3	NKK.4			CVL.4
RRR.4	HQS.3			TRL.3	KKD.4			ESE.4
ISR.4	RKK.3			IIN.3	ETS.4			
PPS.4	LKL.3			GKK.3	LRN.4			
	ISR.3			RRK.3	CSI.4			
	SVM.3			TDF.3	FKK.4			
	LKV.3			FNF.3	KKN.4			
	SDQ.3			RNN.3	APG.4			
	LKR.3			KKK.3	SSK.4			
	QNV.3			RRH.3	RRR.4			
	DKI.3			IRF.3	SFR.4			
	KNK.3			TRR.3	INF.4			
	VGH.3			GRK.3	CVI.4			
	RHH.3			LLH.3	PSN.4			
	CQL.4			LKI.3	SIK.4			
	VAW.4			LQN.3	LQK.4			
	PSH.4			DSD.3				
	MLR.4			SSN.3				
	MEK.4			KKL.3				
	FFS.4			FKK.3				
	CAI.4			KKN.3				

**Figure 4:**
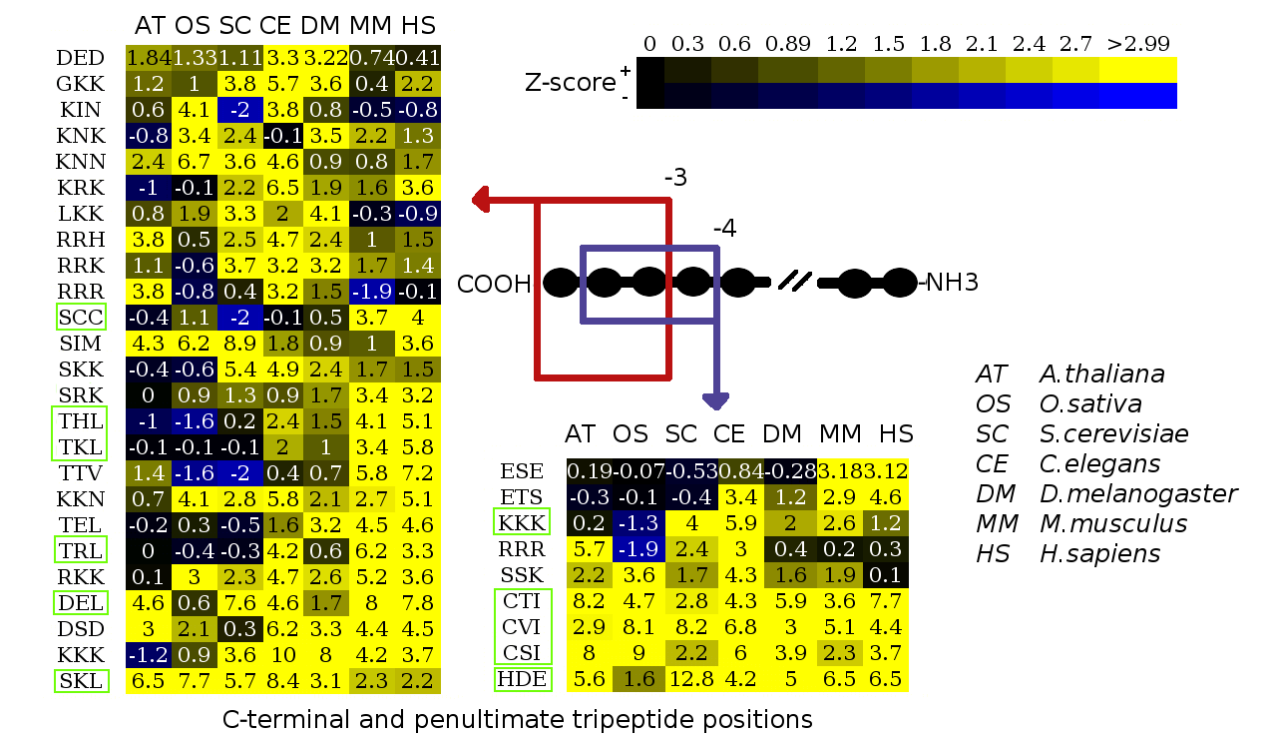
**Heatmap of SOCTs intersected across all genomes examined**. SOCTs present in at least two species and occurring in at least 10 genes in each proteome represented in two blocks of heatmapped z-scores. Positions for the extreme terminal end (-3) and one position in (-4) are shown on the left and right respectively. SOCTs of interest are sorted in increasing significance row-wise with columns listing the species. Tripeptides matching characterized consensus sequences are highlited. Generated with *Heatmapper *[55].

To examine SOCT co-occurrence across all 7 eukaryotic species, the SOCTs were filtered for statistical prevalence (z ≥ 3) in at least 2 genomes and for presence in at least 10 proteins within a species. This later stipulation was introduced to remove rare tripeptides possessing significant z-scores due solely to their genome-wide infrequency and not their terminal abundance. A total of 37 SOCTs emerged at the terminal and second-to-last carboxy positions (see Fig 4). To our knowledge, all reported C-terminal anchored motifs reported in the literature are readily identifiable in these data (Fig 4). These are the peroxisomal targeting PTS1 signal, the ER retrieval/retention signals, Caax box prenylation signals and the Rab protein prenylation motif variant. In addition, several SOCTs match to the PTS1 consensus sequence (ACGST/HKLNR/ILMY*) identified by Mullen et al. [5]; and several variants of the Caax box motif were present [1,5,30].

In total, 35% of the multi-species tripeptide signals occurring in the last two terminal positions matched well characterized C-terminal anchored peptide motifs in the literature. As well as known C-terminal signals, a variety of uncharacterised and potentially functionally important motifs were identified. These motifs may represent, as of yet, unidentified sorting signals but may also represent components of generic C-terminal biology ranging from structural strategies to protein-protein interaction and post-translational modification motifs. For a complete list of identified SOCTs the reader is referred to the supplemental data [see Additional file 6].

## Discussion

The amino and carboxyl termini of proteins are critical components, uniquely positioned to fill a variety of roles in protein biology. Our study has focused on the prediction and identification of novel protein motifs dependent upon C-terminal proximity for proper function. Characterized protein motifs known to function in this manner are largely involved with protein sorting and lipidation [1]. Using integrative genomics and active filtering at the level of sequence and gene family, we have been able to successfully predict a variety of CTAMs and their consensus variants in 7 eukaryotic genomes.

Of all resulting SOCTs, the peroxisomal targeting signal SKL was most prominent (see Table 1, 2; Fig 4). Curiously however, SKL was significantly represented in all species save mammals. Several other PTS1 consensus signals were present in the results and significant only in mammals. These are TKL, THL, TRL and SRK (TRL is also significant in *C. elegans)*. Although these motifs have been demonstrated as conforming to the PTS1 consensus [5], it is unknown if their statistical significance represents a true functional PTS1 signal in mammals or possibly a functional preference for Thr among mammalian PTS1 signals. The addition of functional annotation and protein-protein interaction data could help prove or disprove both of these possibilities.

Given the efficiency with which our analysis was capable of identifying existing C-terminally anchored protein sorting signals, several SOCTs represented across species and within the results of *A. thaliana *were examined for their potential for targeting sufficiency. Unfortunately, none of the SOCTs tested (KNN, KPF, KRR, DSD, SDSD, SDSDSD) using C-terminal GFP fusions exhibited differential sub-cellular localization from an EGFP:AAA control during transient assays in *A. thaliana *and *N. benthamiana *(data not shown). However, other components of a low-level C-terminal protein grammar, such as structural strategies, protein-protein interaction or post-translational modification may be responsible for the high motif frequencies observed in these particular SOCTs.

The terminal tripeptide DSD was highly significant in all species save the proteomes of rice and yeast and similar in significance level to SKL (see Fig. 4). Moreover, 45% of all proteins possessing a DSD motif in all proteomes examined also possessed the terminal sequence of SDSD. Although Ser-Asp repeats did not seem to play a role in targeting, anti-GFP immunoblotting against constitutively expressing GFP and GFP:SDSDSD transgenic *A. thaliana *seedlings showed a slowed migration of a GFP:SDSDSD fusion protein [see Additional file 4]. This preliminary result suggests a potential PTM on the SDSDSD sequence. It is interesting to note that there is a high tendency for proximal serine and acidic residues in proteins possessing the DSD SOCT. Likewise, there are 11 significantly represented serine acidic tripeptides occurring within the terminal 3 positions across all species. The phosphorylation of the terminal DSD in the tumour suppressing protein p53 is known to influence its ability to bind and linearly diffuse along DNA [31]. Similarly, the serine-acidic high mobility group I (HMG1) domains that occur in the C-terminus of HMG proteins, are known to affect both DNA binding and protein stability [32]. HMG proteins dHMGD and dHMGZ both possess the *H. sapiens *SOCT ESE. Also of potential interest are the DSD-6 in RNA polymerase II and ESD-8 in topoisomerase II alpha of *H. sapiens*. Modification of these residues have also been shown to influence DNA binding and protein stability in their respective proteins [33,34]. Although any similarity between these examples and the DSD SOCT itself is uncertain at best, they are nonetheless interesting considering approximately one quarter of DSD possessing proteins in *A. thaliana *are functionally annotated (Gene Ontology) as nucleic acid binding [see Additional file 3]. It does not appear that the prevalence of DSD is a result of an underlying primary nucleotide sequence preference, as the codons in DSD possessing proteins roughly match the codon preferences for each species. However, DSD does conform to the consensus sequences for the di-acid ER export signal, caspase cleavage recognition signals and the CKII consensus sequence [1], the latter two of which are frequently C-terminal focused. In any case, it would seem that serine-acidic motifs in the C-termini of eukaryotes are likely functionally active and potentially fulfill a variety of roles such as PTM and signal transduction.

An interesting, albeit unexpected result within the SOCT intersections of *A. thaliana *and *O. sativa*, was the presence of a highly conserved sequence (FSDENPNA-4) proximal to the Caax motif in a group of iso-prenylated plant metalloproteins [see Additional files 5 and 6]. Although the highly divergent nature of the family prevented this motif from being filtered out, its proximity to a prenylation signal makes this conserved region of special interest. Recent bioinformatics has suggested that residue biases in hydrophobicity exist in sequences proximal to many Caax boxes [30]. Does the Caax proximal sequence play a role in the successful prenylation of these proteins? Based on its degree of conservation, it would appear to be critical to this metalloprotein family's function. There is evidence that the prenylation reaction performed by farnesyltransferase is dependent upon a metal ion nucleophile provided by a metalloprotein cofactor [35].

There is a strong presence of Lys among many of the uncharacterised tripeptides at the terminal end of the 7 species "C-terminome". These include: KNK, KNN, KKN, KRK, RRK, KKK, GKK and LKK (see Fig 4). In 2003, Chung et al. proposed the C-terminal lysine preference in yeast was due to capping preferences in protein stability [22]. Di-basic or C-terminal basic residues regulating a proteins trafficking have also been reported. Both the nucleotide receptor P2X7 and the GluR6 kainate receptor possess basic C-terminal tails in which the mutation or deletion of basic residues from the terminus motif disrupted proper protein targeting [36,37]. Another basic motif involved in targeting is the di-Lys motif at -4, which assists in protein sorting via retrieval of proteins to the ER [1]. The possibility exists that these basic SOCTs reflect a loose consensus for the core residues of a protein-protein interaction domain specific to a class of subcellular targeting chaperones.

Overall several intriguing patterns in peptide compositional preferences have been identified. Although the present analysis focuses on the C-terminus, it should be noted that an N-terminal examination was run in parallel and similar biases were observed at the N-terminus [see Additional file 7]. A couple observations of note in the N-terminal statistically over-represented tripeptides are the high prevalence of alanines at the penultimate position. This agrees with bias tendencies seen in other studies and corresponds to strategies in protein half-life as dictated by the N-end rule [38]. A very prominent motif was the MASS motif, which has been implicated in transcript stability at the codon level [39]. Data obtained from studies at both termini are available on the paper's web-site [40] and are offered to the public for further study [see also Additional files 6 and 7].

## Conclusion

Several properties of the C-terminal class of anchored motifs make them attractive for *ab initio *motif discovery. Since the carboxyl group provides a point of reference, C-terminal anchored peptides should appear among peptide frequencies calculated at distinct C-terminal positions [18]. Likewise, their low information content allows for a direct examination of short peptides (tripeptides in this study). These factors greatly simplify probability calculations, as complex considerations for motif size and positioning can be excluded. Additionally, as characterized C-terminal anchored motifs are known to function across a variety of proteins and families, the removal of tripeptide counts from large C-terminal conserved protein families should not affect the significance score of a true motif, but rather should reduce false positives arising from family-specific homology. Indeed, this filter proves most effective in improving the signal-to-noise ratio, as seen in Figure 2B. This integration of C-terminal tripeptide statistics with protein family information, in combination with simple sequence masking and comparative genomics, was successfully applied to the prediction of C-terminal specific motifs *ab initio*. Given our success in predicting known motifs, the likelihood of novel yet undefined motifs present in the results seems likely. However, among the previously known motifs identified, the majority are widely prevalent with strong significance values. This suggests that any novel uncharacterised signals present in the data may function more specifically or subtly than other confirmed CTAMs present in the analysis.

Since the C-terminus is a frequent site for protein regulation and is often utilized in recombinant protein experiments, it would seem that C-terminal peptide function will continue to increase in relevance as our knowledge of its biological importance progresses. The novel SOCTs identified in our analysis may represent C-terminal peptide motifs functioning in biological roles ranging from protein sorting, post-translational modification or capping and structural strategies. However, based on the prominence of known targeting signals and the lack of novel SOCTs with a distinct pattern, any protein sorting motifs that remain to be characterized are likely to be conserved to a small number of protein families, exhibit species-specific functionality or possess a considerable degree of degeneracy. Overall, our results appear to depict a highly accurate representation of the statistical topography of the "C-terminome" and the methodology could be adapted to protein motif prediction efforts in the protein interior.

## Methods

Prior to statistical analysis, each predicted proteome was clustered into protein families using the shell script *famMCL *and masked for simple sequence stretches using the program *seg *[25]. Mean and standard deviation values, derived from randomized sets for each species, were used to calculate individual z-scores for each possible tripeptide at each position from the extreme C-terminal position to 100 residues in from the carboxyl group. This yielded a comprehensive collection of 776,000 C-terminal z-statistics (8000 possible tripeptides × 97 positions: -100 to -3). Results were then intersected between species proteomes, to test for the presence of SOCTs (z ≥ 3) in at least 2 species and tripeptide presence in at least 10 different proteins within each respective proteome.

### Datasets

Translated datasets for each species were obtained in fasta format. All datasets were downloaded from NCBI with the exceptions of *A. thaliana *which was obtained from TAIR and *O. sativa*, which was downloaded from TIGR. *O. sativa *was downloaded in conjunction with a list of accessions corresponding to transposable elements. This list was then used to filter out transposable elements from the protein summary file with a short shell script.

*A. thaliana *downloaded from TAIR as ATH1_pep_cm_20040228. [41]

*C. elegans *by chromosome translated faa. [42]

*D. melanogaster *by chromosome translated faa. [43]

*H. sapiens *by protein summary. [44]

*M. musculus *by protein summary. [45]

*S. cerevisiae *by chromosome translated faa file. [46]

*O. sativa *by protein summary. [47]

*O. sativa *transposable element list by accession for the filtering of above. [48]

### Proteome randomization

To generate a collection of randomized fasta sequences, the program *fastarand *was written in the C programming language [49]. Given a fasta formatted file, *fastarand *will create an equal size fasta file on a sequence by sequence basis using one of three randomization models.

1) shuffle the amino acids within each protein in the file

2) generate each sequence based on the amino acid frequencies in the entire proteome

3) resample nmers from the query protein until an equal length protein is reached

The user is able to specify how many randomized proteomes are to be created with a commandline flag (set to 100 in our analysis). Model 3 using 3mers was employed in our study.

### Protein family classification

Each species proteome was clustered into gene families using the short shell script *famMCL*. *famMCL *should compile on any POSIX based system and depends upon a functional installation of NCBI standalone blast [50], and the *MCL *clustering algorithm [51]. *famMCL *and its supporting documentation are available under the GPL [52].

*famMCL *performs an all-by-all *BLASTP *of the provided proteome using the concise output format option of the NCBI standalone *BLAST *program (-m 8). All bit scores for individual protein comparisons are parsed from the *BLAST *results to produce an *MCL *format matrix that is submitted to the *MCL *clustering algorithm. The resulting *MCL *output is then parsed to generate lists of protein families by accession and corresponding cluster number. A *BLAST *similarity cutoff of E ≤ 1e-10 and the default *MCL *granularity were used. Our strategy for implementation resulted in little to no variation in cluster composition in successive runs over the recommended range of *MCL *granularity settings and fluctuations in cluster size and composition within *famMCL *were found to be primarily dependent upon selection of the E-value cutoff. As an all-by-all genome *BLAST *is computationally intensive, it was performed on an openSSI parallelized cluster of workstations running Debian GNU Linux at the Botany Bioinformatics Cluster in the Department of Cell & Systems Biology, University of Toronto.

### The SOCT pipeline

The program *tripepper *was written in the C programming language to determine mean and standard deviation background statistics for respective tripeptides from 100 *fastarand *randomized proteomes. The PERL script *cluster adjuster *was then used to calculate position specific tripeptide counts for each simple sequence masked proteome and adjust these counts by subtracting duplicate tallies arising from members of a common gene family as derived from *famMCL*. These programs collectively constitute the SOCT pipeline software [53] and return a comprehensive set of z-statistics for all 8000 possible tripeptides at each position from the terminal end (-3) to 97 positions in from the carboxy-terminus (-100). Protein family information is used to reduce tripeptide counts artificially inflated due to homologous gene clusters, using a penalized z-statistic as calculated by:

Σ_i_Σ_j _Z_λ _= (*k*_ij _- *x*_ij _- λ_ij_)/*s*_ij_

where i is a tripeptide permutation between AAA and YYY, j is the position in from the C-terminus (-3 to -100), *k *is the number of counts for the tripeptide in the masked proteome, λ is the number of duplicate tripeptide counts due to a common gene family and *x *and *s *are the mean and standard deviation respectively for occurrences of tripeptide_ij _across 100 randomized proteomes.

The program *tripepper *was given a *fastarand *generated directory of randomized proteomes and a corresponding proteome with stretches of simple sequences masked out. Masked proteomes were created by running *seg *at default settings [25]. Note that the randomized sets are not masked and that the masked proteome as produced by *seg *is used to replace the original proteome read by tripepper. Protein clusters as determined by *famMCL *were then input to the Perl program *cluster adjuster*, which uses the *tripepper *results and the masked proteome to adjust total tripeptides counts by the number of common family occurrences and produce a set of final penalized z-scores.

### Data integration, filtering and visualization of raw data

Comprehensive z-statistics generated for each eukaryotic proteome were processed using common UNIX shell scripting tools (e.g.: *grep*, *sed*, *awk*) to identify all significant tripeptides (z-score ≥ 3) occurring in at least 2 species and present in at least 10 genes in each proteome. These data were then analysed using the open source plotting program *gnuplot *[54] or fed to the web-based application *Heatmapper *[55] to generate a z-score based visual heatmap of all intersecting SOCTs (see Fig 4).

## Abbreviations

CTAM – Carboxy-terminal anchored motif

SOCT – Statistically Over-represented Carboxy-terminal Tripeptide

PTM – Post-translational modification

GPL – Gnu General Public Licence

## Authors' contributions

SRC conceived of the concept, RSA performed the analyses under the supervision of SRC and NJP. RSA wrote the manuscript, which was edited by RSA, NJP and SRC. All authors read and approved the final manuscript.

## Supplementary Material

Additional File 7 All filtered N-terminal tripeptide z-statistics for all species Click here for file

Additional File 2 Background reduction from TE filtering (*O. sativa*) Click here for file

Additional File 1 Background reduction in randomization models (*A. thaliana*) Click here for file

Additional File 5 famMCL generated protein families in all species Click here for file

Additional File 6 All filtered C-terminal z-statistics for all species Click here for file

Additional File 3 GO annotation for DSD possessing genes in *A. thaliana* Click here for file

Additional File 4 Immunoblotting against EGFP:SDSDSD transgenic *A. thaliana* Click here for file
