# Supplementary figures and images for: C-terminal motif prediction in eukaryotic proteomes using comparative genomics and statistical over-representation across protein families
Source: BMC Genomics. 2007 Jun 26;8:191. doi: 10.1186/1471-2164-8-191 (PMC1929074; doi:10.1186/1471-2164-8-191)

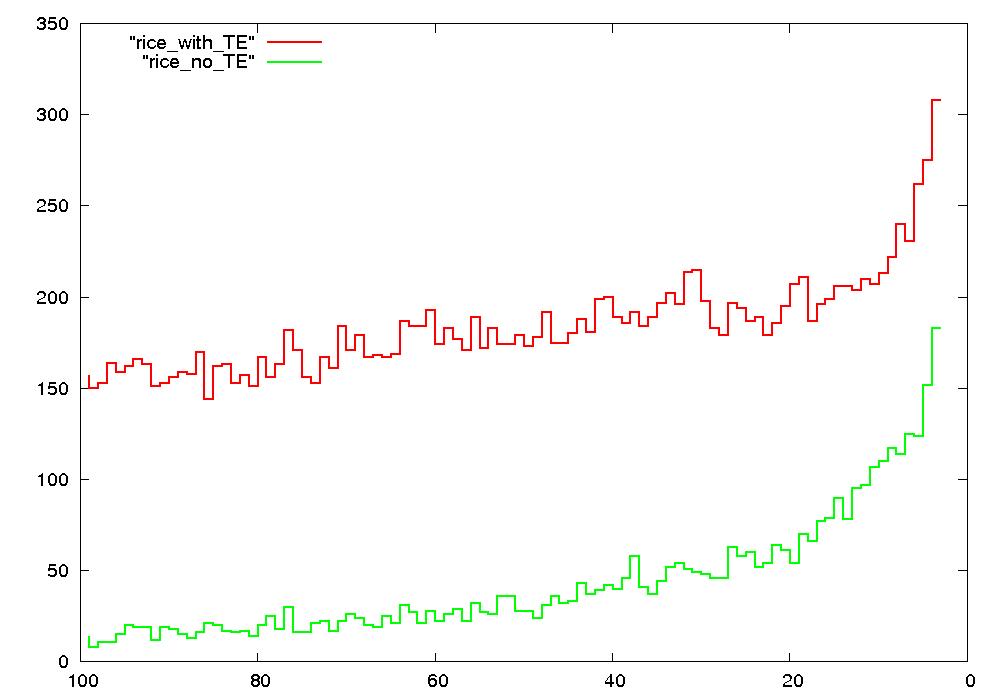

Supplement: Additional File 2 — Background reduction from TE filtering (O. sativa) [file 1471-2164-8-191-S2.png]

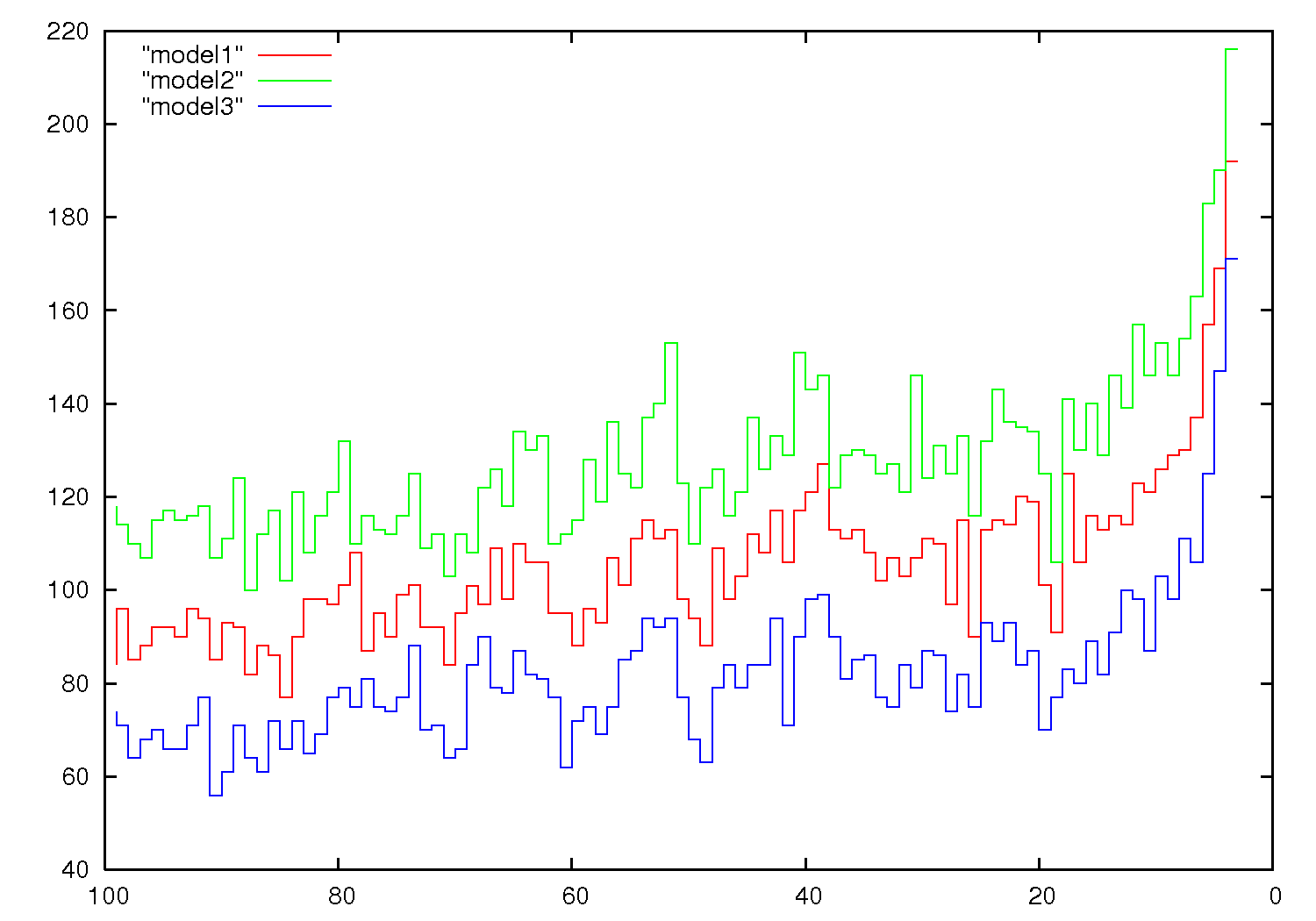

Supplement: Additional File 1 — Background reduction in randomization models (A. thaliana) [file 1471-2164-8-191-S1.png]
