# Supplementary material for: C-terminal motif prediction in eukaryotic proteomes using comparative genomics and statistical over-representation across protein families
Source: BMC Genomics. 2007 Jun 26;8:191. doi: 10.1186/1471-2164-8-191 (PMC1929074; doi:10.1186/1471-2164-8-191)
Supplement: Additional File 3 — GO annotation for DSD possessing genes in A. thaliana [file 1471-2164-8-191-S3.pdf]

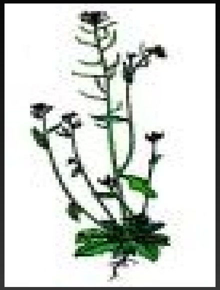

GO annotation for DSD-cooh proteins

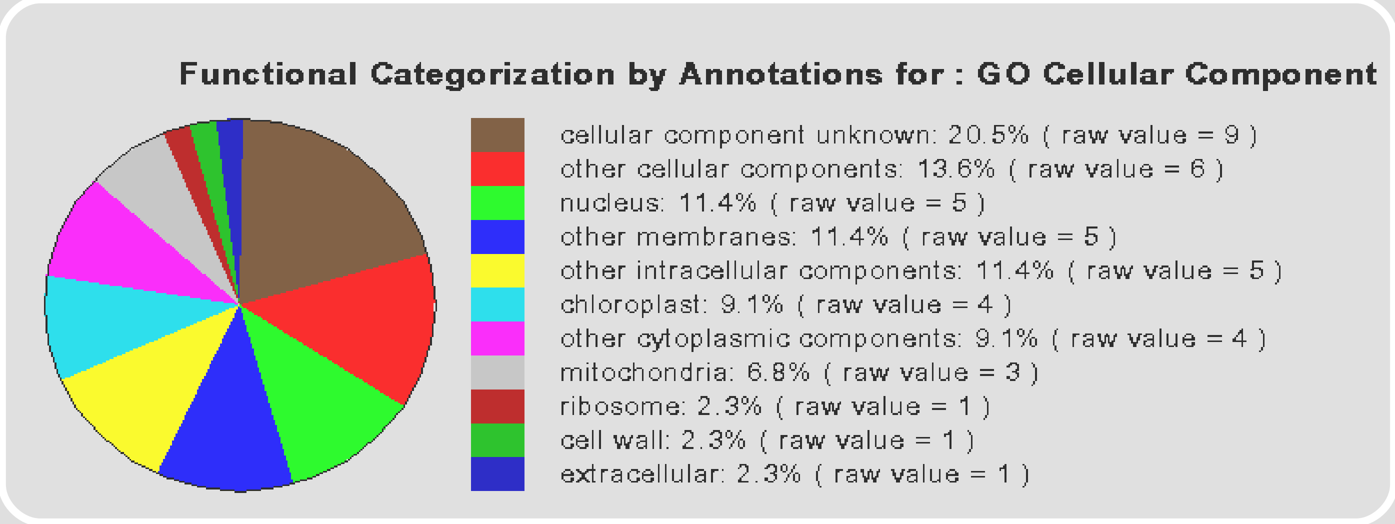

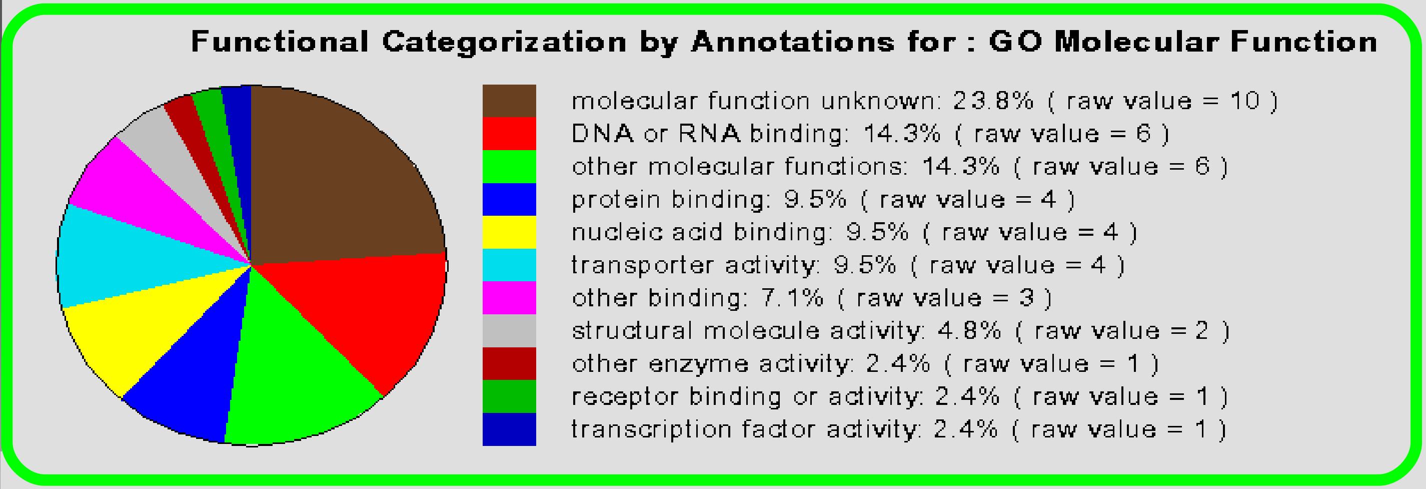

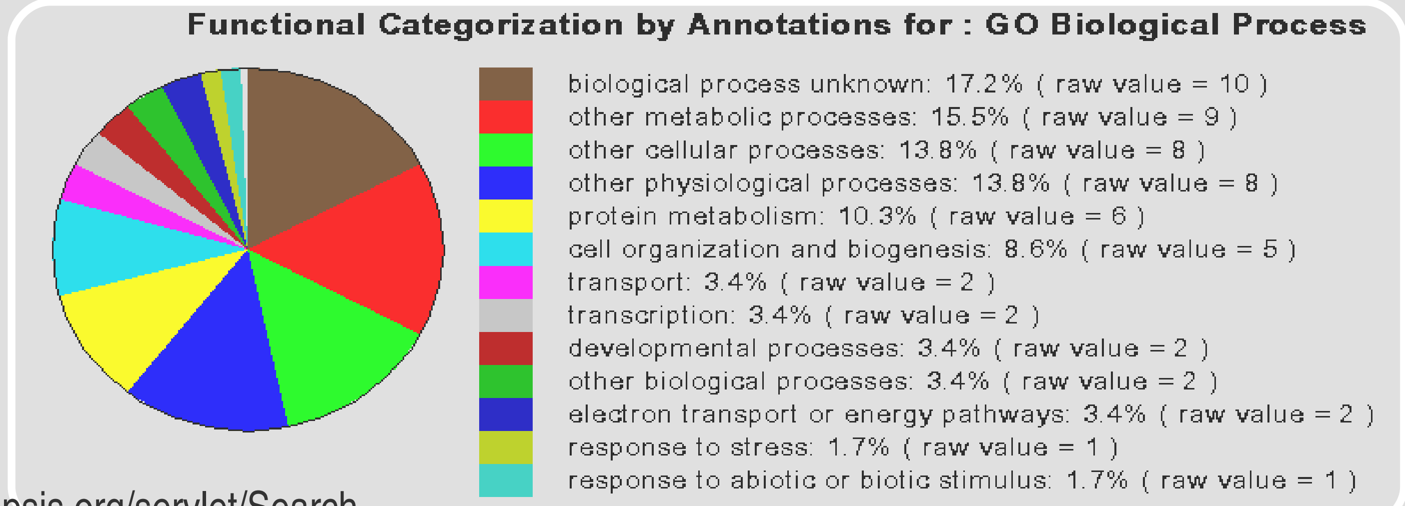

http://www.arabidopsis.org/servlet/Search
