# Supplementary material for: C-terminal motif prediction in eukaryotic proteomes using comparative genomics and statistical over-representation across protein families
Source: BMC Genomics. 2007 Jun 26;8:191. doi: 10.1186/1471-2164-8-191 (PMC1929074; doi:10.1186/1471-2164-8-191)

C-terminal fusion of SDSDSD peptide to EGFP in A.*thaliana*

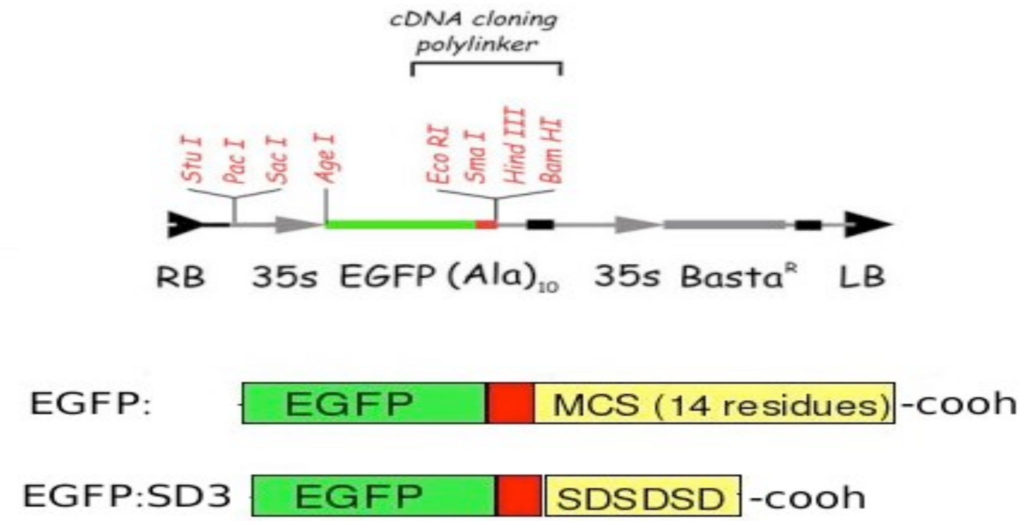

+ - + -

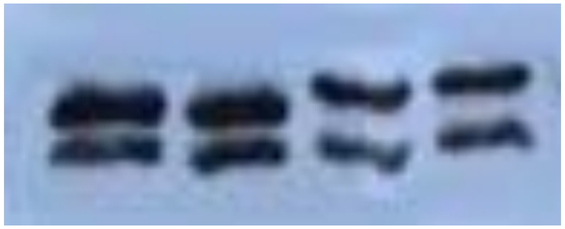

GFP SD3

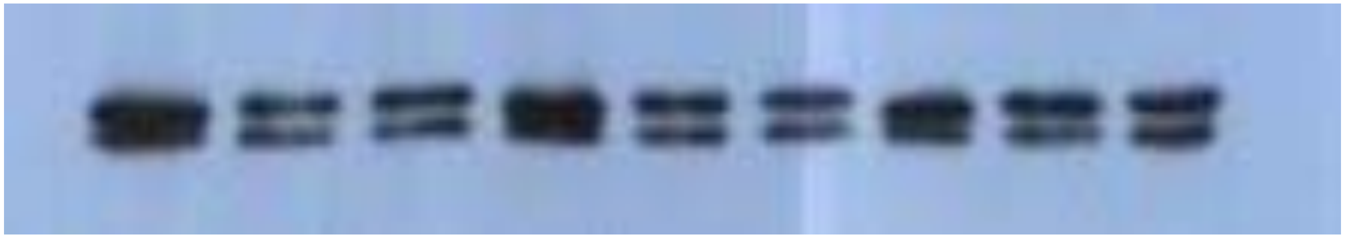

calf-intestinal phosphatase	GFP	SD3	SD3	GFP	SD3	SD3	GFP	SD3	SD3
		+	-		+	-		+	-

A c-terminal fusion of a putative *A.thaliana* dehydrin to EGFP.

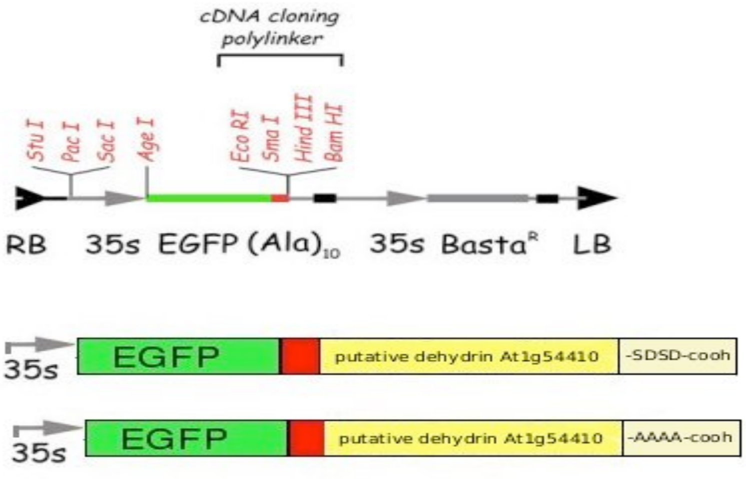

D4

D4-SD2ΔA4

D4 D4-SD2ΔA4

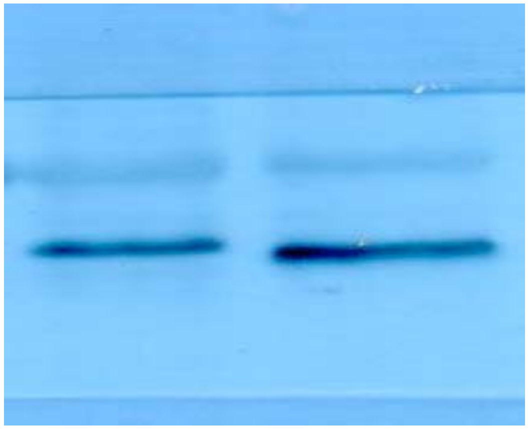

D4 D4-SD2ΔA4

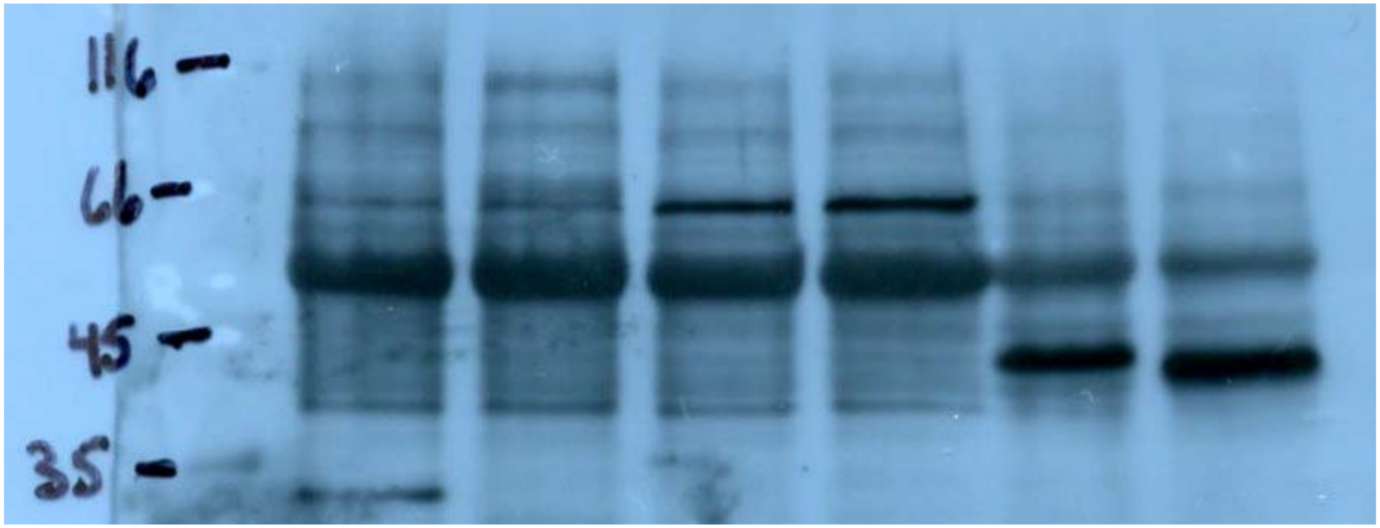

Supplement: Additional File 4 — Immunoblotting against EGFP:SDSDSD transgenic A. thaliana [file 1471-2164-8-191-S4.pdf]
